# Prediction mapping of human leptospirosis using ANN, GWR, SVM and GLM approaches

**DOI:** 10.1186/s12879-019-4580-4

**Published:** 2019-11-13

**Authors:** Ali Mohammadinia, Bahram Saeidian, Biswajeet Pradhan, Zeinab Ghaemi

**Affiliations:** 1 GIS Division, Faculty of Geodesy and Geomatics, K. N. Toosi University of Technology, Tehran, Iran; 2 The Centre for Advanced Modelling and Geospatial Information Systems (CAMGIS), Faculty of Engineering and IT, University of Technology Sydney, Sydney, NSW 2007 Australia; 3 Department of Energy and Mineral Resources Engineering, Sejong University, Choongmu-gwan, 209 Neungdong-ro, Gwangjin-gu, Seoul, 05006 Republic of Korea

**Keywords:** Leptospirosis, GIS, ANN, GWR, SVM, GLM, Machine learning, Prediction

## Abstract

**Background:**

Recent reports of the National Ministry of Health and Treatment of Iran (NMHT) show that Gilan has a higher annual incidence rate of leptospirosis than other provinces across the country. Despite several efforts of the government and NMHT to eradicate leptospirosis, it remains a public health problem in this province. Modelling and Prediction of this disease may play an important role in reduction of the prevalence.

**Methods:**

This study aims to model and predict the spatial distribution of leptospirosis utilizing Geographically Weighted Regression (GWR), Generalized Linear Model (GLM), Support Vector Machine (SVM) and Artificial Neural Network (ANN) as capable approaches. Five environmental parameters of precipitation, temperature, humidity, elevation and vegetation are used for modelling and predicting of the disease. Data of 2009 and 2010 are used for training, and 2011 for testing and evaluating the models.

**Results:**

Results indicate that utilized approaches in this study can model and predict leptospirosis with high significance level. To evaluate the efficiency of the approaches, MSE (GWR = 0.050, SVM = 0.137, GLM = 0.118 and ANN = 0.137), MAE (0.012, 0.063, 0.052 and 0.063), MRE (0.011, 0.018, 0.017 and 0.018) and R^2^ (0.85, 0.80, 0.78 and 0.75) are used.

**Conclusion:**

Results indicate the practical usefulness of approaches for spatial modelling and predicting leptospirosis. The efficiency of models is as follow: GWR > SVM > GLM > ANN. In addition, temperature and humidity are investigated as the most influential parameters. Moreover, the suitable habitat of leptospirosis is mostly within the central rural districts of the province.

## Background

Since the discovery of leptospira in the body of Japanese mine workers over a hundred years ago, human leptospirosis has been treated as a “neglected tropical disease” worldwide [1]. Reports of World Health Organization show that annual incidence rate of leptospirosis per 100,000 people varies from 0.1 to 1 in temperate regions and 10–100 in humid regions and over 100 in tropical areas. Global report of the disease reveals that over 1 million severe cases take place annually with approximately 60,000 fatalities [2]. As a Zoonotic disease, it occurs in tropical and sub-tropical areas with high humidity [3]. This disease is caused by leptospira bacteria which live in the urine of mammals such as rodents [4]. Human infection from leptospirosis occurs through direct or indirect contact with infected animals or environment [5]. Several contributing factors are contemplated for the incidence of leptospirosis including geographical location with frequent rainfall and floods, adjacency to mammal reservoirs and human activities [6]. One of the most important reasons of leptospirosis mortality is its resemblance to other diseases such as influenza and dengue fever [7]. Indeed, underestimating its infectiousness and loss of timely diagnosis give rise to fatality [8].

Rafyi and Magami in 1968 confirmed the first report of human leptospirosis in Iran, but no definite report has been made about the current status of human leptospirosis distribution in the country [9]. Human leptospirosis, an endemic disease in Caspian region, is more widespread in Gilan Province because of humid and wet climate [3]. In addition, high population densities of rural districts, farmlands (often paddy fields) and fishing activities help propagate the prevalence of leptospirosis in Gilan. Amongst provinces, the annual incidence rate of leptospirosis in Gilan is always the highest. In this region, most farmers keep domestic animals in their houses and irrigate their farms using river resources, where the population of leptospirosis-contaminated rodents is abundant [9]. Hence, modelling and predicting leptospirosis will help policy makers to better understand the disease, prioritise regions and budget for early prevention or treatment and provide accurate planning. It will help the government policy makers ease the burden of medical and health care expenditure on the province.

Several studies were made on modelling leptospirosis worldwide [10–13]. Many studies elucidated the effect of drivers such as precipitation [14, 15], temperature [16, 17], humidity [18, 19], elevation [20] and vegetation [10, 21] on the distribution of leptospirosis because its prevalence highly depends on environmental factors. However, most studies focused on clinical aspects of the disease and animal type of leptospirosis. Based on literature review and to the best of our knowledge, papers rarely worked on spatial modelling and predicting human leptospirosis utilising Geographical Information System (GIS) and its approaches [11, 12].

GIS is a powerful tool that its capabilities have been already proven in various fields of studies such as disease [22–24] and environment [25–27]. In disease problems, GIS can play a major role in showing how the disease propagates and finding the parameters that affect its prevalence [28]. The advantages of GIS have been proven in developed countries, but it is rarely employed for health issues in developing countries such as Iran [29, 30].

Given that the heterogeneity relationship between the disease and effective parameters, some methods should be utilized to consider heterogeneity [31]. Geographically weighted regression (GWR) is a common approach that can solve the heterogeneity by considering variability of coefficients in diverse locations across the study area [32]. An advantage of GWR is considering the location of parameters as input to improve spatial prediction capability and reduce heterogeneity effect. GWR is an efficient approach for modelling in various fields of study [33–35], especially disease modelling and predicting. However, GWR is a linear method that cannot consider the nonlinear behaviour of the phenomenon. Owing to high capability in solving nonlinear problems, Artificial Neural Network (ANN), a widely used approach in disease prediction, is selected to predict leptospirosis disease [36–39]. Another approach used in this study is General Linear Model (GLM), which is a statistical model commonly used in modelling and predicting diseases [40]. It utilizes the polynomial regression to investigate the relationship between dependent and independent variables [41]. Also, SVM, a supervised classifier, is used as a novel machine learning method which can be used for classification and in regression analysis [42]. The SVM classifier takes a set of input dataset and predicts the class of each input data which is used in various medical issues [30, 43] .

This study aims to model and predict human leptospirosis in Gilan Province of Iran, using capabilities of GWR, GLM, SVM and ANN approaches. Background section provides knowledge about leptospirosis and the reasons of its prevalence based on previous studies. Methods section explains how data are prepared and asserts fundamentals about utilized approaches. Results section presents the results of models. Discussion section interprets data ally with analysing the information which can be obtained from the results of the models in detail. The final section describes the conclusions of the study and indicates future work.

## Methods

### Study area

**Fig. 1:**
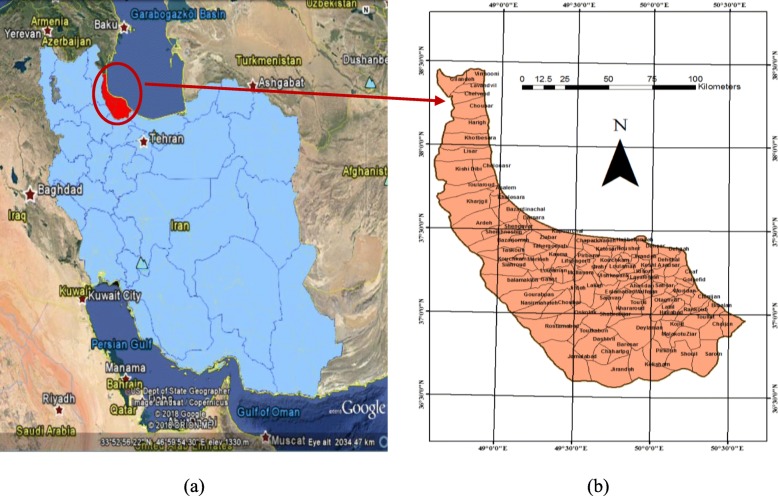
**a** Location of Gilan Province, (**b**) Rural districts of Gilan

Gilan, a northern province of Iran, ranks second in rice cultivation. Figure 1 depicts the geographical location of Gilan at 48°53′–50°34′ longitude and 36°34′–38°27′ latitude. It consists of approximately 2.531 million inhabitants, 107 rural districts and 14,042 km^2^ area. It stretches across Alborz Mountains with dense forests in the south (highlands) and Caspian Sea in the north (lowlands). In this study, modelling is performed at the rural district level, and the centroids of the rural districts are considered as the base level for analysis. These centroids are selected as the points to which all parameters are allocated. Notably, the centroids are the geometric centres of polygons of the rural districts. The mean, maximum and minimum area of the rural districts are 129,253,376 m^2^, 441,566,882 m^2^ and 113,055,500 m^2^, respectively.

### Data acquisition and preparation

The input parameters utilized in this study are disease, climate, topography and vegetation data collected from relevant organisations in Iran (Ministry of Health and Meteorology Agency) from 2009 to 2011. The population data of rural districts used in this study are gathered from the National Centre of Statistics of Iran, and these data are updated every 5 years by this organisation across the country. The latest updated population data at the rural district level of Gilan, which included the population size of different divisions of the country separately, are used in this study. The data in 2009 and 2010 are used for modelling, and the models are assessed by the data in 2011. All data are prepared and integrated using ArcGIS 10.2 and Microsoft Excel 2010 for further analysis. To avoid very large or small weights, the input data are normalised between [0,1] using Eq. (1) [44].

$$ Normalized\ (x)=\raisebox{1ex}{$\left({x}_i-{x}_{min}\right)$}\!\left/ \!\raisebox{-1ex}{$\left({x}_{max}-{x}_{min}\right)$}\right. $$

where *x*_*i*_ denotes the input parameter; *x*_*min*_ and *x*_*max*_ are minimum and maximum values of *x*_*i*_, respectively.

### Disease data

**Fig. 2:**
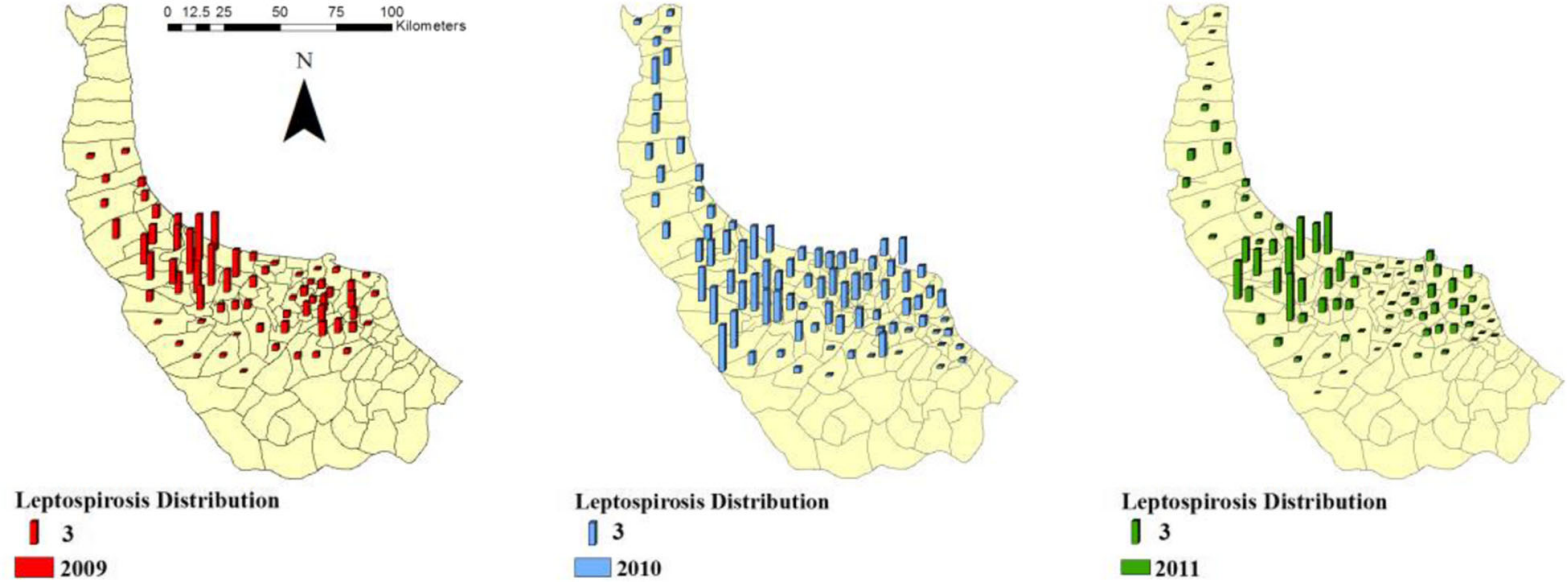
Leptospirosis distribution of Gilan at the rural district level in 2009–2011

All villages in Iran are covered by the well-founded National Health Care Network (NHCN), which is sponsored by National Ministry of Health and Treatment of Iran. The disease data (positive results of ELISA^1^ blood test of patients) are gathered from database of NHCN and Health Centres (HC) of Gilan. The spatial distribution of the disease throughout the study area is illustrated in Fig. 2.

Incidence rate measures the frequency of disease occurrence in the population over a specified time. The major advantage of calculating incidence rate is the omission of the effect of population on disease prevalence across the study area. To eliminate the effect of population on results, incidence rate is calculated using Eq. (2):

2 $$ \mathrm{Incidence}\ \mathrm{Rate}=\frac{number\ of\ Leptospirosis\ cases}{Popula\mathrm{t} ion\  at\  risk}\ast \mathrm{10,000} $$

### Climate data

**Fig. 3:**
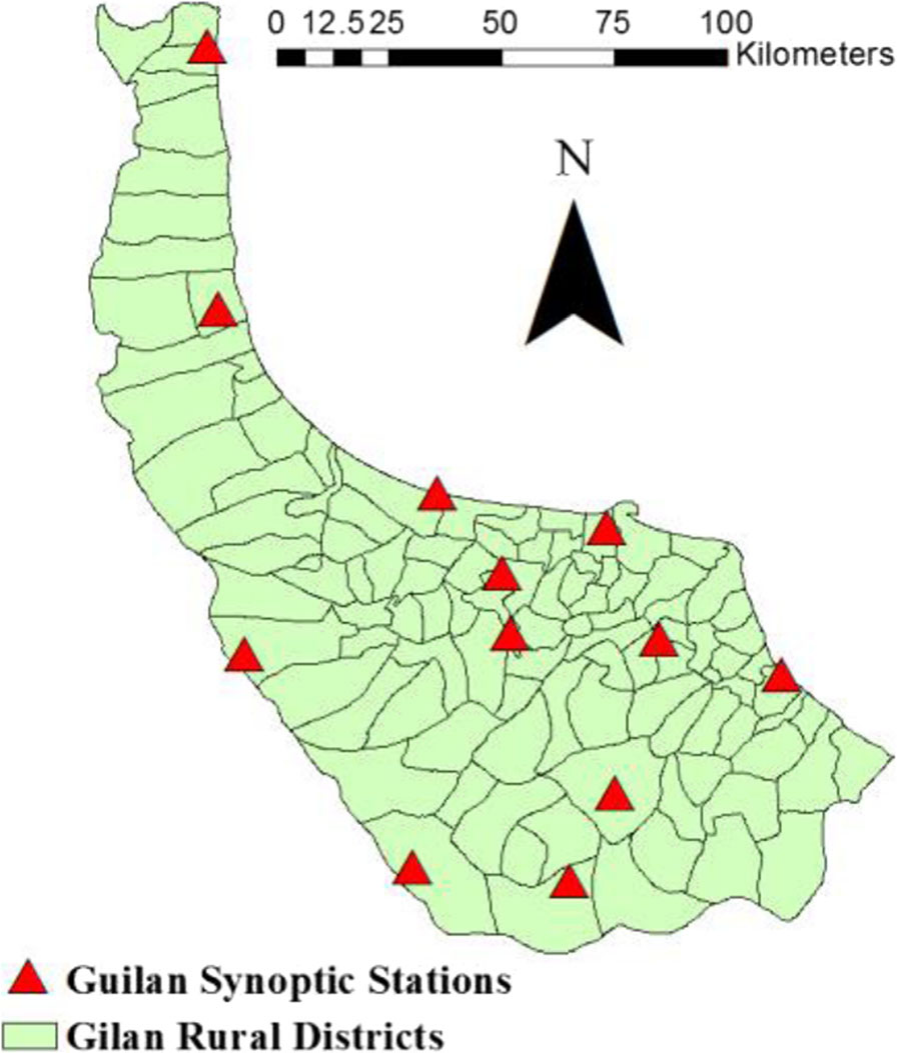
Distribution of 12 synoptic climate stations across Gilan

Temperature (degree Celsius), humidity (percentage) and precipitation (millimeter) are gathered from 12 synoptic climate stations of Gilan in Excel format (.xlsx) (Fig. 3).

**Fig. 4:**
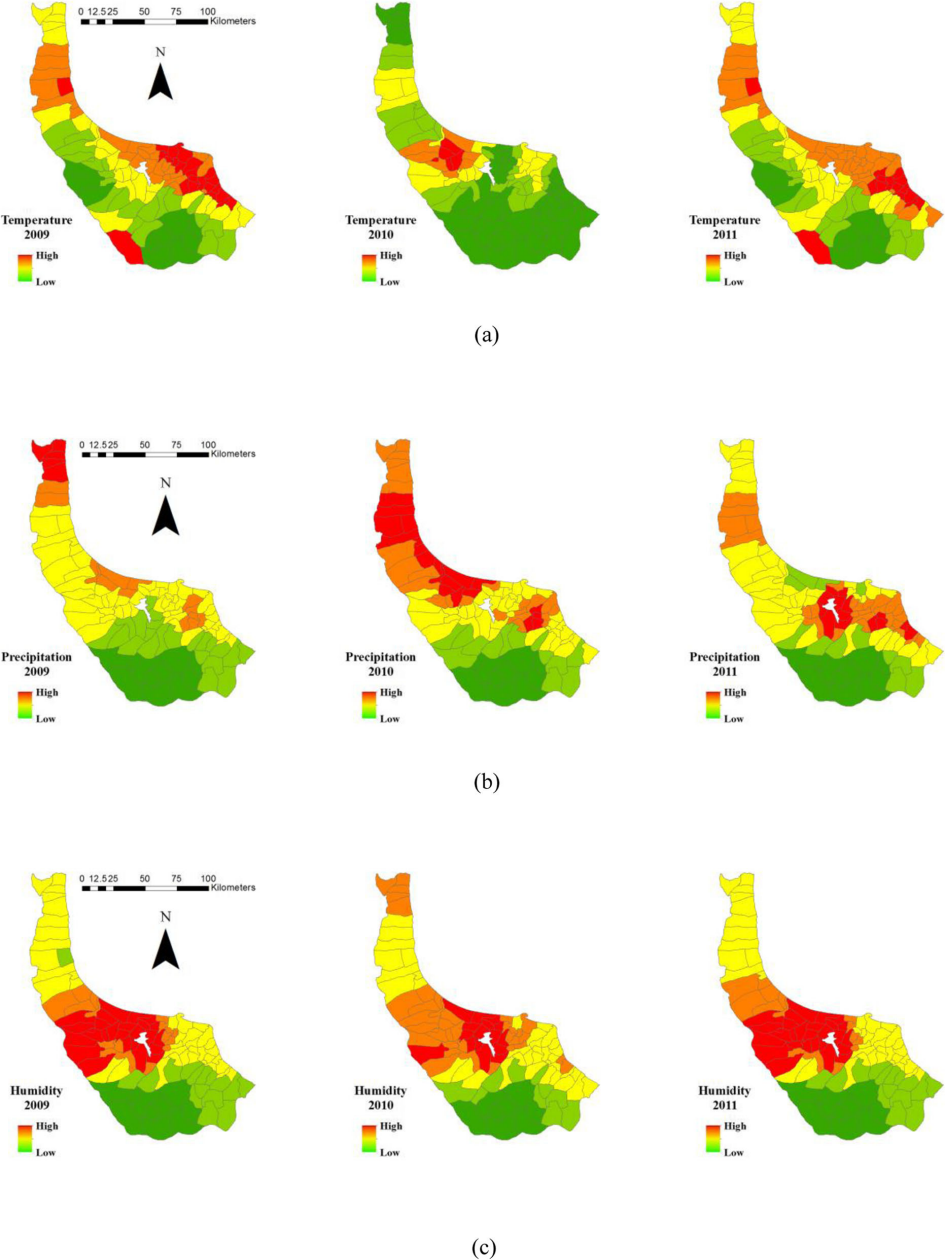
**a** Average temperature, (**b**) precipitation, (**c**) and humidity at rural districts of Gilan in 2009–2011

Given that the climate data are collected from the meteorological stations and the limited number of these stations across the study area, a continuous surface of the climate parameters is produced utilising IDW^2^ interpolation method. The obtained maps are demonstrated in Fig. 4.

### Topographic and vegetation data

Gilan shows remarkable topographic variations with almost 3700 m altitude difference between the lowest and highest locations and average altitude of 1800 above sea level. Elevation continually decreases from south to north. Owing to the significant variability of elevation, climate and vegetation differ across the study area. The elevation map is obtained from NASA^3^‘s 90 m resolution SRTM^4^data. All parameters such as elevation are assigned to the centroids of rural districts for further analysis. ArcGIS software tool ‘Extract to Points’ is employed, and the elevation data are assigned to the centroids.

**Fig. 5:**
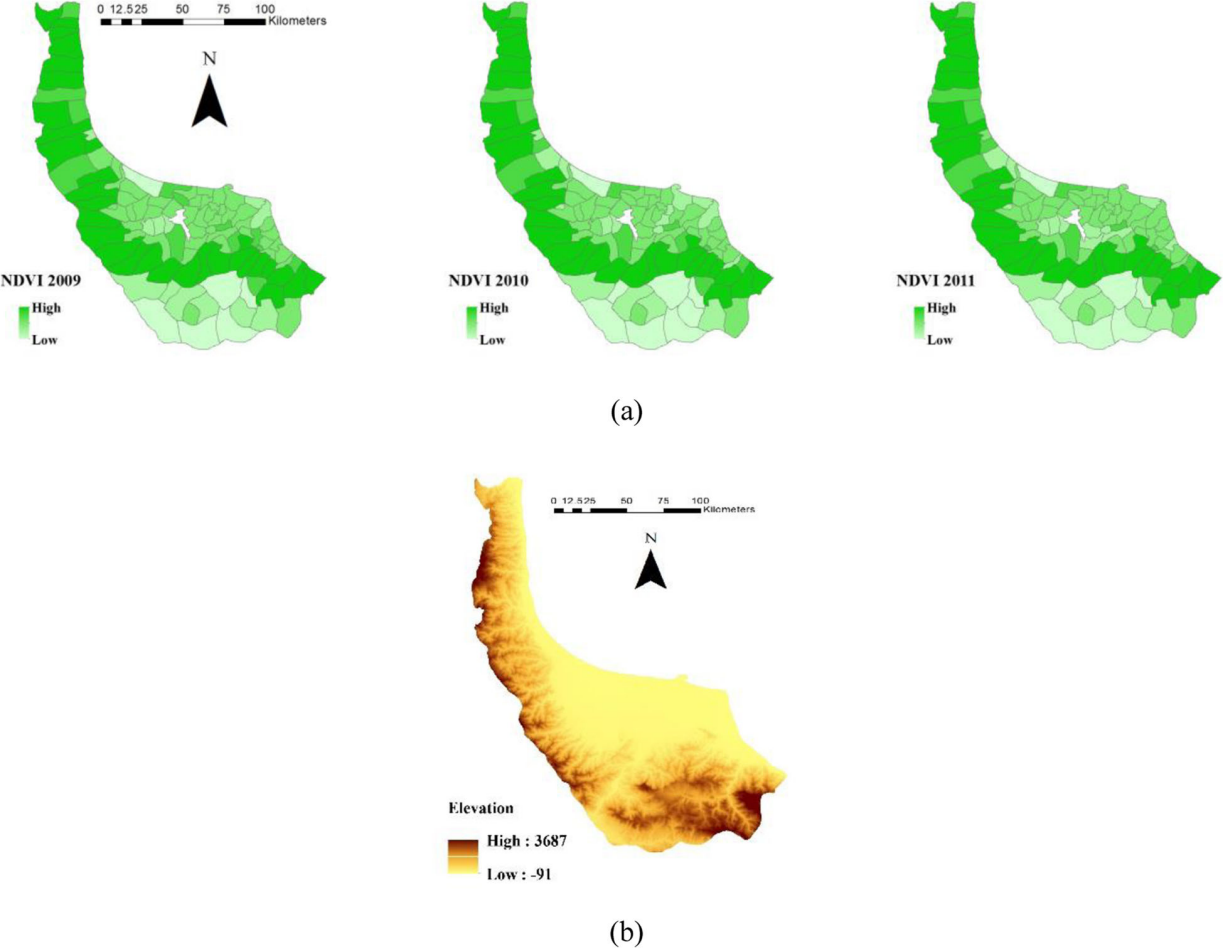
**a** NDVI and (**b**) Average elevation of rural districts of Gilan in 2009–2011

Vegetation is another environmental factor which influences leptospirosis vector directly or indirectly [10]. To investigate the effect of vegetation at the rural district level, Normalised Difference Vegetation Index (NDVI) is used in this study. This process is performed using the satellite images of the Gilan and the capabilities of ENVI^5^ software, a well-known software in image processing. Satellite images of MODIS^6^ during 2009–2011 are used to extract NDVI via ENVI software. Their period is 16-day ally with 250 m spatial resolution. The satellite images are mosaicked, and the NDVI index of study area is subsequently calculated and used as the vegetation parameter in the study. The vegetation values of all rural districts are allocated using the calculated NDVI. Elevation data and variability of NDVI in 2009 and 2011 are presented in Fig. 5.

All parameters and their characteristics are presented in Table 1.

**Table 1:** Input parameters and their characteristics

Parameter	Type	Data Description	Unit
Disease data	Output of model	Positive reported cases of human leptospirosis across Gilan province	Number
Temperature	Input of model	Monthly average temperature of rural districts	Centigrade
Precipitation	Input of model	Monthly average rainfall of rural districts	Millimetre
Humidity	Input of model	Monthly average humidity of rural districts	Percentage
Elevation	Input of model	Average height of rural districts	Meter
Vegetation	Input of model	Average NDVI of rural districts	Without unit

### GWR

GWR presented by [45] is the most important regression approach in spatial modelling. The general equation of this approach is expressed as follows (Eq. (3)):

3 $$ {Y}_j={B}_0\left({U}_j,{V}_j\right)+{\sum}_k{B}_k\left({U}_j,{V}_j\right)\ {X}_{jk}+{\upvarepsilon}_j $$

where j = 1,2,…,n shows the number of rural districts, *Y*_*j*_ is the incidence rate of leptospirosis in rural district *j*, (*U*_*j*_, *V*_*j*_) denotes the geographic location of rural district *j*, *B*_*i*_ is the local coefficient of parameter *k*, *X*_*jk*_ is the value of input parameter in rural district *j*, ε_*j*_ is the error value, and *B*_*k*_ is obtained from minimising Eq. (4):

4 $$ {B}_0\left({U}_j,{V}_j\right)=\sum \limits_{k=1}^n{W}_{jk}{\left({Y}_j-{B}_0\left({U}_j,{V}_j\right)-\sum \limits_{k=1}^p{B}_k\left({U}_j,{V}_j\right)\ {X}_{jk}\right)}^2 $$

where *W*_*jk*_ is distance decay function for location *j*. Three distance decay functions are applicable in GWR model, namely, Poisson, Gaussian and Logistic. In this study, Gaussian function (Eq. (5)) is used due to its higher efficiency [46]:

5 $$ {W}_{jk}=\exp \left(-{d}_{jk}^2/{b}^2\right) $$

where *d*_*jk*_ is the spatial distance between rural district *i* and *k*, and *b* identifies the kernel bandwidth. Three bandwidth selection criteria, including AIC (Akaike Information Criterion), CV (Cross Validation) and BIC (Bayesian Information Criterion), and two kernels (fixed and adaptive) are available in modelling by GWR [45].

### GWR model for leptospirosis prediction

To predict leptospirosis, a model is established based on environmental parameters utilising GWR approach. Five parameters, including temperature, precipitation, humidity, elevation and vegetation, in 2009 and 2010 together with disease data are used as inputs of the model. The model is used for predicting of leptospirosis in 2011.

According to the description of methods and input parameters, the GWR model is formulated as Eq. (6):

6 $$ {Y}_j={B}_0\left({U}_j,{V}_j\right)+{\mathrm{B}}_{temp}{X}_1\left({U}_j,{V}_j\right)+{\mathrm{B}}_{prec}{X}_2\left({U}_j,{V}_j\right)+{\mathrm{B}}_{hum}{X}_3\left({U}_j,{V}_j\right)+{\mathrm{B}}_{elev}{X}_4\left({U}_j,{V}_j\right)+{\mathrm{B}}_{Veg}{X}_j\left({U}_5,{V}_j\right) $$

where *Y*_*j*_ denotes the incidence rate of leptospirosis (the independent parameter), B_*temp*_, B_*prec*_, B_*hum*_, B_*elev*_ and B_*Veg*_ are the correlation coefficient values of input parameters, *X*_1_, *X*_2_, *X*_3_ and *X*_4_ are the values of dependent parameters in a definite rural district, and (*U*_*j*_, *V*_*j*_) denotes the location of rural district *j*.

Fixed and adaptive kernel functions are applicable for the GWR model. Fixed kernel considers a constant bandwidth (distance to neighbour in metre) across the study area, which is the main deficiency of this kernel, whereas adaptive kernel applies variable and appropriate bandwidths (number of neighbours) in each rural district according to the number of neighbours [47]. In addition to type of kernel, defining bandwidth selection criteria is necessary in the GWR model. Three bandwidth criteria of AIC, CV and BIC are available. Adaptive kernel and AIC criteria are utilized in this study due to better performance [48]. Notably, all steps are performed using GWR 4.0 software.^7^

### ANN

ANN is a nonlinear model that focuses on determination of dependence between input and output parameters by simulating highly connected processing units (neurons) of human nervous system [49]. It consists of three layers including input, hidden and output, and it is composed of weighted connections between the inputs and outputs [50]. A major characteristic of ANN is its capability to learn for solving complex problems [51]. The other advantage of ANN is proper description of nonlinear dependences. However, the black box mechanism is its major shortcoming [52].

A particular form of ANN is Multilayer Perceptron (MLP) which is created by multiple layers of nodes in a directed graph [53]. MLPs are Feed-Forward Neural Networks (FFNN) that stream information in one direction from the input to the output layer. MLPs are the most popular FNNs due to efficient training processes [54].

**Fig. 6:**
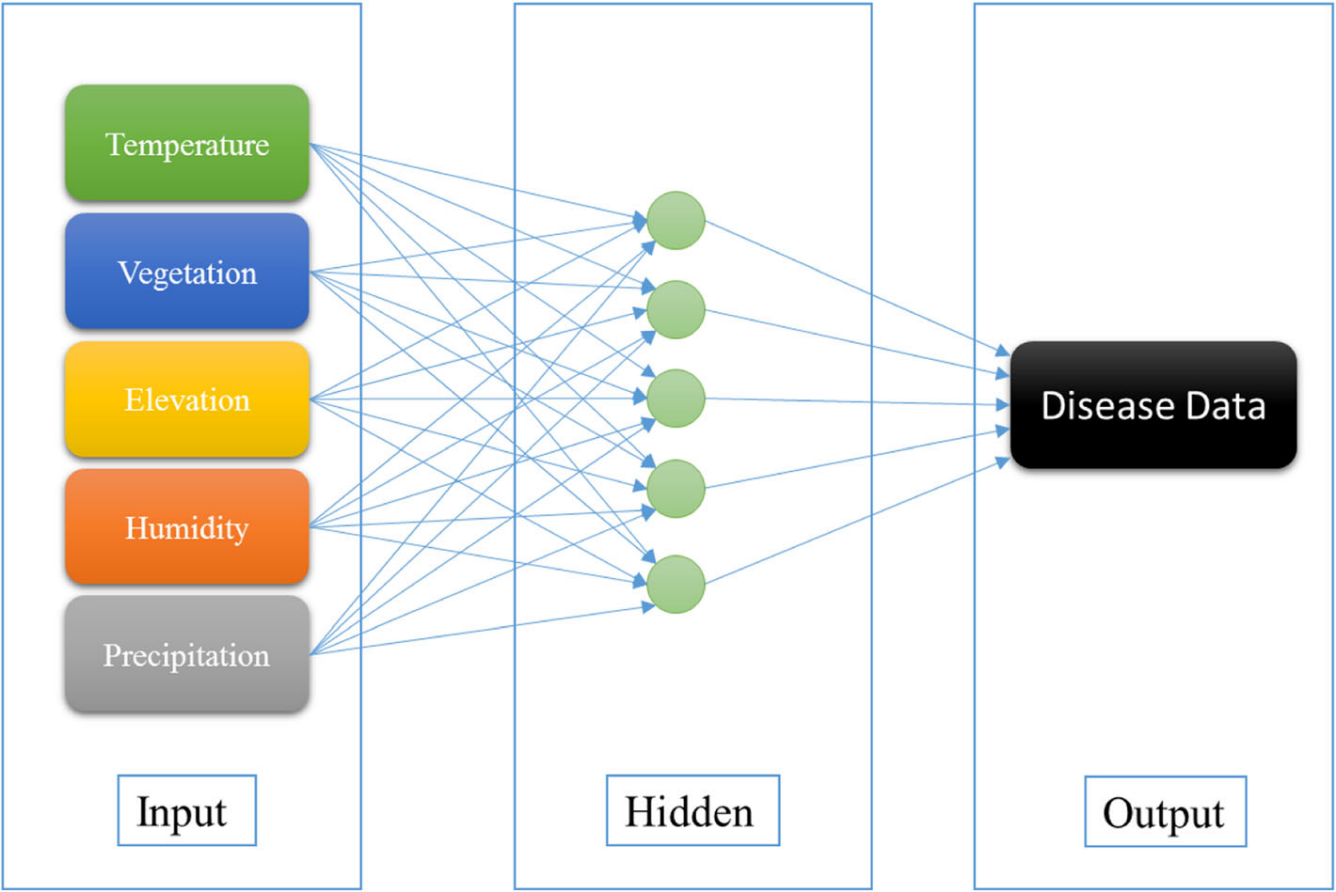
Structure of MLP used in this research

In ANN, input data should be normalised before feeding to the model because different data with diverse ranges should be mapped into a similar range. Training data which adjust the weights of neurons and decline the model bias are also important in modelling using ANN [55]. Data training has several algorithms, and Levenberg–Marquardt algorithm is a popular one [56]. After training data, test data should be utilized to evaluate the performance of the network. Figure 6 exhibits the structure of MLP used in this research.

### ANN model for leptospirosis prediction

MLP, a class of FFNN is utilized for leptospirosis prediction. MATLAB 2018 is used for MLP implementation. According to the trial and error approach (Additional file 1), one hidden layer is selected to be utilized in this study. The final MLP architecture consists of five nodes in input layer, including temperature, precipitation, humidity, elevation and vegetation, one hidden layer with five nodes and one node in output layer, which presents the incidence rate of leptospirosis. Data of 2009 and 2010 and Leungberg–Marquard algorithm are used for training the model to predict the disease in 2011. Weights are randomly initialised, and the threshold of the training process is considered when the error difference of two consecutive runs of the model is negligible. Notably, after running ANN under such condition (reaching a negligible difference of two consecutive runs), the maximum number of epochs is 36. Total sample points are 969 for 2009 and 2010 in which 290 samples are selected as validation set. The learning rate, which is acquired using trial and error approach, is 0.01.

### SVM

SVM, first introduced by Vapnik [57], is a supervised classifier based on the statistical theory. In a linear situation, the basic SVM tries to maximize the distance between closest samples of binary classes by creating optimal hyperplanes [57]. However, most of the problems in real world do not behave in linear manner. In order to deal with non-linear datasets, SVM utilizes kernel functions to map data into higher dimensional space in which the data is linearly separable [58].

Consider the input data as {*x*_*i*_, *x*_2_, …, *x*_*i*_} named vectors and their corresponding labels as *y*_*i*_ ∈ {−1, +1}, SVM constructs hyperplanes which separate positive labels from negative ones. Equations (7) and (8) are used to investigate the label of data in non-linear situation [59]:

7 $$ f(x)=\mathit{\operatorname{sign}}\left\{\sum \limits_{i=1}^l{\alpha}_i{y}_ik\left({x}_i,{x}_j\right)+b\right\} $$

8 $$ \mathrm{Subject}\ \mathrm{to}\ \mathrm{the}\ \mathrm{constraints}:\sum \limits_{i=1}^l{\alpha}_i{y}_i=0\ \mathrm{and}\ 0\le {\alpha}_i\le C\  for\  all\ i $$

Where b is the bias, *K*(*x*_*i*_*x*_*j*_) is the kernel function and *α*_*i*_ denotes the Lagrange’s multiplier which can be calculated by maximizing eq. (9). *C* is regularization constant which balances the maximization of sample distances and model error [60].

9 $$ \operatorname{Maximize}\ {\sum}_{i=1}^l{\alpha}_i-\frac{1}{2}{\sum}_{i=1}^{;}{\sum}_{j=1}^l{\alpha}_i{\alpha}_j{y}_i{y}_jK\left({x}_i{x}_j\right) $$

### SVM model for leptospirosis prediction

In order to apply SVM model, input data are categorized into 5 classes (very low, low, moderate, high, and very high classes). The Data of 2009 and 2010 is used to train the SVM model and it is utilized to predict leptospirosis in 2011. Because SVM is a binary classifier, it cannot be directly used for a multiclass problem. In order to perform a multiclass classification using a binary classifier, one-against-all method can be used to divide each multiclass classification into groups of binary classifications [57]. In this study, 5-bainry SVMs are constructed (5 is the number of classes) in which, each binary classifier separates one class from the rest of the classes. Another vital step in running an SVM model is the selection of its parameter (C) and the type of kernel function [59, 61]. Leave-one-out cross-validation method [62] is applied on training dataset to select Parameter C and the value of 2 is obtained as the best value in this study. The most common kernel functions have been used in previous studies are the linear, polynomial, and Radial Basis Function (RBF) [63]. Therefore, in order to determine the best kernel function, these functions are compared in this study and the output result is presented in Table 2. As it is shown in this table, RBF could obtain more accurate result in this study. Java programming language is used to implement SVM in this study.

**Table 2:** Efficiency of different kernel functions

Kernel Function	R^2^
Linear	0.63
RBF	0.80
Polynomial (degree 2)	0.65
Polynomial (degree 3)	0.50

### Sensitivity analysis

ANN and SVM function as a black box, so investigating the relative importance of input parameters is not possible. However, sensitivity analysis can be used to examine the contribution of input parameters in modelling and predicting [64]. To perform sensitivity analysis, one parameter is excluded from the model in each run, and the effect of that parameter on model performance is determined based on the evaluation criteria [65]. A larger decrease indicates greater influence of the parameter.

### GLM

The Generalized Linear Model is one the most common statistical approach identified for prediction mapping [66]. GLM assumes a relationship between the dependent variable and different independent variables given by (Eq. (10)):

10 $$ E\ (y)=\mu =\sum \limits_{j=1}^p{X}_j{B}_j $$

where ***E*** (***y***) is the value of dependent variable y, ***X***_***j***_ indicates j^th^ independent variables regarding to ***p*** covariates to be estimated and ***B***_***j***_ is the j^th^ coefficient.

### GLM model for leptospirosis prediction

GLM model is established based on the input variables in which the variables do not change locally in spite of GWR model. In this study the following model is used as the GLM model for prediction of leptospirosis:

11 $$ Ln\ (A)= Ln\ \left({B}_0\right)+{B}_1{X}_1+{B}_2{X}_2+\dots +{B}_p{X}_p $$

where *Ln (A)* is log of disease data, *X*_*j*_, j^th^ independent variables (*j = 2, ..., p*) and *B*_*j*_, j^th^ coefficients of variables (*j = 0, ..., p*). The ordinary least-squares estimates are calculated to obtain Maximum-likelihood estimates for GLM which performs like a multivariate analysis. All implementation of this approach is done using SPSS software version 23.

### Spatial autocorrelation

Spatial autocorrelation is useful for analysing and examining randomness of residuals [67]. Moran’s *I* is commonly used for checking spatial autocorrelation and cluster detection which ranges between − 1 and 1 (Eq. (12)) [67]:

$$ {I}_i={Z}_i\sum \limits_1^n{W}_{ij}{Z}_j $$

12 $$ {Z}_i=\left({Y}_i-\overline{Y}\right)/S $$

where *W*_*ij*_ is the spatial weight between i^th^ and j^th^ provinces; z_i_ and z_j_ are the values of z-score in i^th^ and j^th^ provinces, respectively; Y_i_ is the number of cases for i^th^ province; and S is the sum of all spatial weights. Moran’s I is used to determine the spatial autocorrelation of residuals for investigating the model deficiencies.

### Evaluation

To assess the results of approaches, Mean Square Error (MSE), Mean Absolute Error (MAE), Mean Relative Error (MRE) and R^2^ are employed as Eqs. 13–16.

MSE is the most common statistic for regression evaluation and it is defined as follow (Eq. (13)) [68]:

13 $$ MSE=\frac{\sum_{i=1}^n{\left({y}_i-{\hat{y}}_i\right)}^2}{n} $$

where *y*_*i*_ is leptospirosis report and *ŷ*_*i*_ is its prediction. For each rural district, it calculates the average square difference between the predictions and actual values. It is useful when we have unexpected values that we should pay attention.

MAE means that all the individual differences are weighted equally in the average. It is calculated using Eq. (14) [68]:

14 $$ MAE=\frac{\sum_{i=1}^n\left|{y}_i-{\hat{y}}_i\right|}{n} $$

The advantage of this statistic is that it is not sensitive to outliers as MSE. We considered the relative errors in each rural district and calculate the mean of it to obtain MRE value. Equation ((15)) represents this statistic [69]:

15 $$ MRE=\frac{\sum_{i=1}^n\left|\frac{y_i-{\hat{y}}_i}{y_i}\right|}{n} $$

Realizing the performance of models is difficult when we use only MSE, MAE and MRE criteria. R^2^ is a metric has the advantage of being scale-free and can solve this issue. Many papers indicate that the range of R^2^ is between 0 and 1. Equation (16) is used to calculate R^2^ [36, 61]:

16 $$ {R}^2=1-\frac{\sum_{i=1}^n{\left({y}_i-{\hat{y}}_i\right)}^2}{\sum_{i=1}^n{y_i}^2} $$

where *y*_*i*_ is the incidence rate in rural district *i*, ${\hat{y}}_i$ is the predicted value, and *n* is the number of rural districts.

## Results

**Fig. 7:**
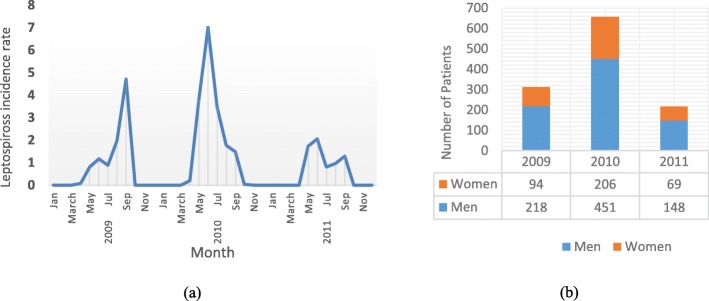
**a** Leptospirosis incidence rate and (**b**) Number of patients according to gender in 2009–2011

According to the database of NHCN, leptospirosis occurs annually in certain months (approximately March to September) and remarkably coincides with the beginning of rice planting and end of harvest season (Fig. 7.a). Reports confirmed that in 2009 (312 cases), 2010 (657 cases) and 2011 (217 cases), 1186 positive cases were reported, and the peak of leptospirosis prevalence occurred in 2010 in Gilan, which is twice as much as last year. Amongst reported cases, 70% of patients were men who are more vulnerable to leptospirosis infection than women (Fig. 7.b).

The calculated correlations of input parameters are shown in Table 3. The table shows the maximum correlation between elevation and temperature (− 0.33), which is consistent with reality (the higher elevation, the lower the temperature); the minimum is between vegetation and temperature (0.11). Variance inflation factor (VIF) is calculated for input parameters, and the results are presented in Table 3. All VIF values are less than 2.71, confirming no severe multicollinearity amongst input parameters.

**Table 3:** Pearson correlation coefficients among parameters

Variable	Criteria	Temperature	Precipitation	Humidity	Elevation	Vegetation	VIF
Temperature	Correlation						2.20
	Significance						
Precipitation	Correlation	0.25^*^					1.17
	Significance	0.002					
Humidity	Correlation	0.28^*^	0.29^*^				1.64
	Significance	0.002	0.003				
Elevation	Correlation	−0.33^*^	−0.31^*^	−0.15**			2.44
	Significance	0.005	0.003	0.077			
Vegetation	Correlation	0.11**	0.26^*^	0.27^*^	−0.12**		2.71
	Significance	0.098	0.003	0.003	0.63		

* Correlation coefficient with 0.05 significance level

** Correlation coefficient with 0.1 significance level

Minimum, maximum, range and standard deviation obtained from GWR model are presented in Table 4, which shows the variability of each parameter in the spatial modelling of leptospirosis.

**Table 4:** Coefficients of parameters using GWR model

Parameter	Min	Max	Range	Std
Temperature	− 109.37	1631.32	1740.69	241.26
Precipitation	− 164.45	292.79	321.64	42.43
Humidity	− 336.93	476.01	812.94	129.89
Elevation	−0.59	0.24	0.83	0.17
Vegetation	−0.15	0.23	0.39	0.09

Table 5 presents the coefficients of input parameters obtained from GLM model. They clarify the impact of each parameter on modelling leptospirosis distribution.

**Table 5:** Coefficients of parameters using GLM model

Parameter	B	Standard Error
Temperature	19.4	28.04
Precipitation	−7.41	6.57
Humidity	13.25	16.01
Elevation	3.80	0.29
Vegetation	−2.87	0.14

The output of sensitivity analysis of ANN and SVM are presented in Table 6 and Table 7. Temperature and humidity are utmost effective parameters of leptospirosis prediction because their removal leads to a decrease in the value of four criteria. On the contrary, removing vegetation and precipitation lead to improving the accuracy of prediction, which shows less effect of both parameters in prediction.

**Table 6:** Results of sensitivity analysis in ANN model

	Temperature	Precipitation	Humidity	Elevation	Vegetation
R^2^	−0.07	0.03	−0.06	−0.02	0.02
MSE	−0.0203	0.0052	−0.0131	−0.0048	0.0032
MAE	−0.0221	0.0011	−0.0118	−0.066	0.0070
MRE	−0.0214	0.0041	−0.0109	−0.0032	0.0041

**Table 7:** Results of sensitivity analysis in SVM model

	Temperature	Precipitation	Humidity	Elevation	Vegetation
R^2^	−0.09	0.03	−0.05	− 0.01	0.01
MSE	−0.0316	0.0021	−0.0276	−0.0062	0.0051
MAE	−0.0298	0.0019	−0.0206	−0.059	0.0062
MRE	−0.0284	0.0026	−0.0266	−0.0028	0.0055

**Fig. 8:**
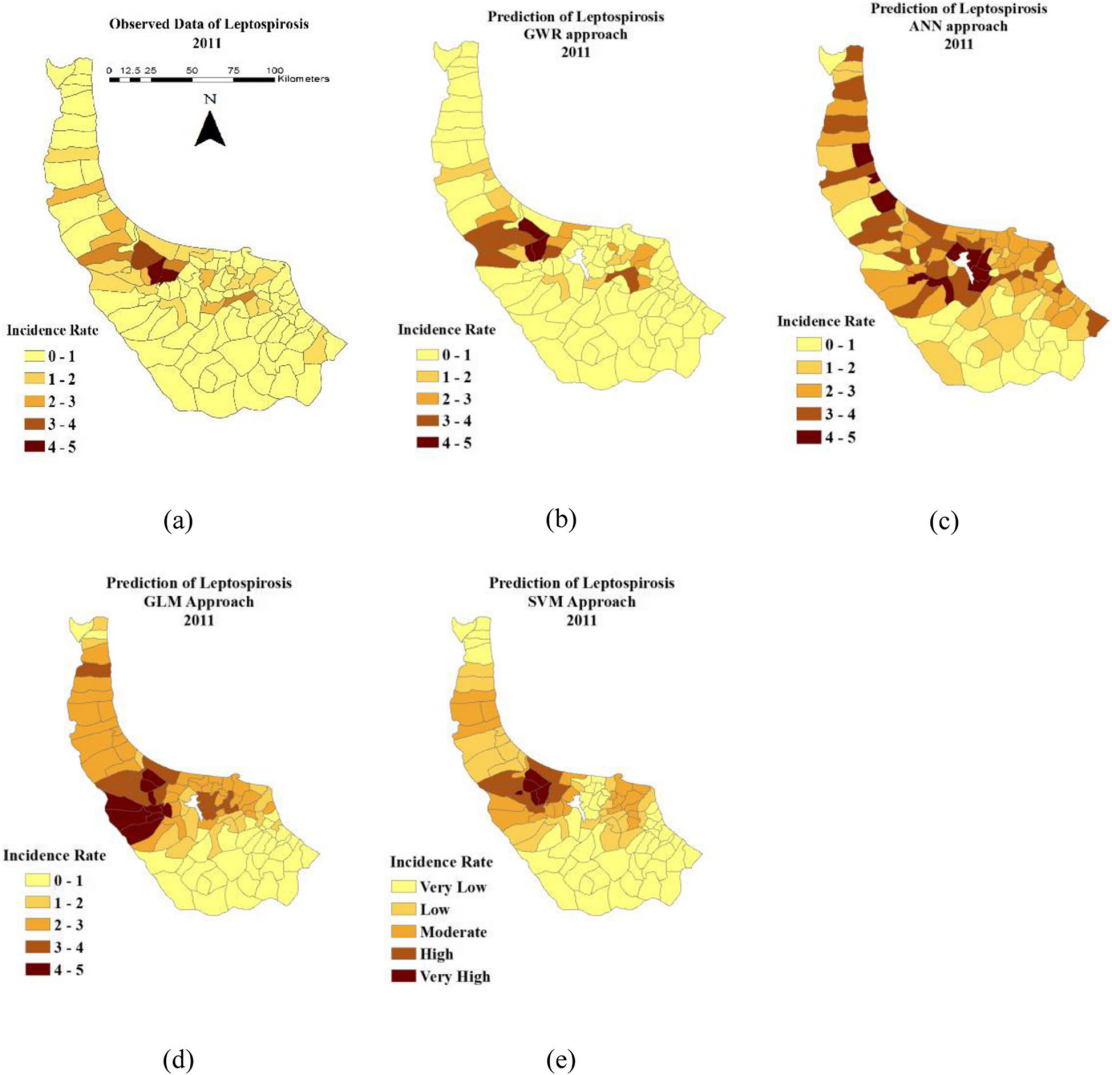
**a** Leptospirosis data of 2011, (**b**) Prediction maps using GWR, (**c**) ANN, (**d**) SVM and (**e**) GLM models

Figure 8.a shows the actual number of leptospirosis disease in 2011. Figure 8.b, 8.c, 8.d and 8.e show the results of GWR, ANN, SVM and GLM prediction in 2011, respectively. The disease rarely occurs in the southeast rural districts.

**Fig. 9:**
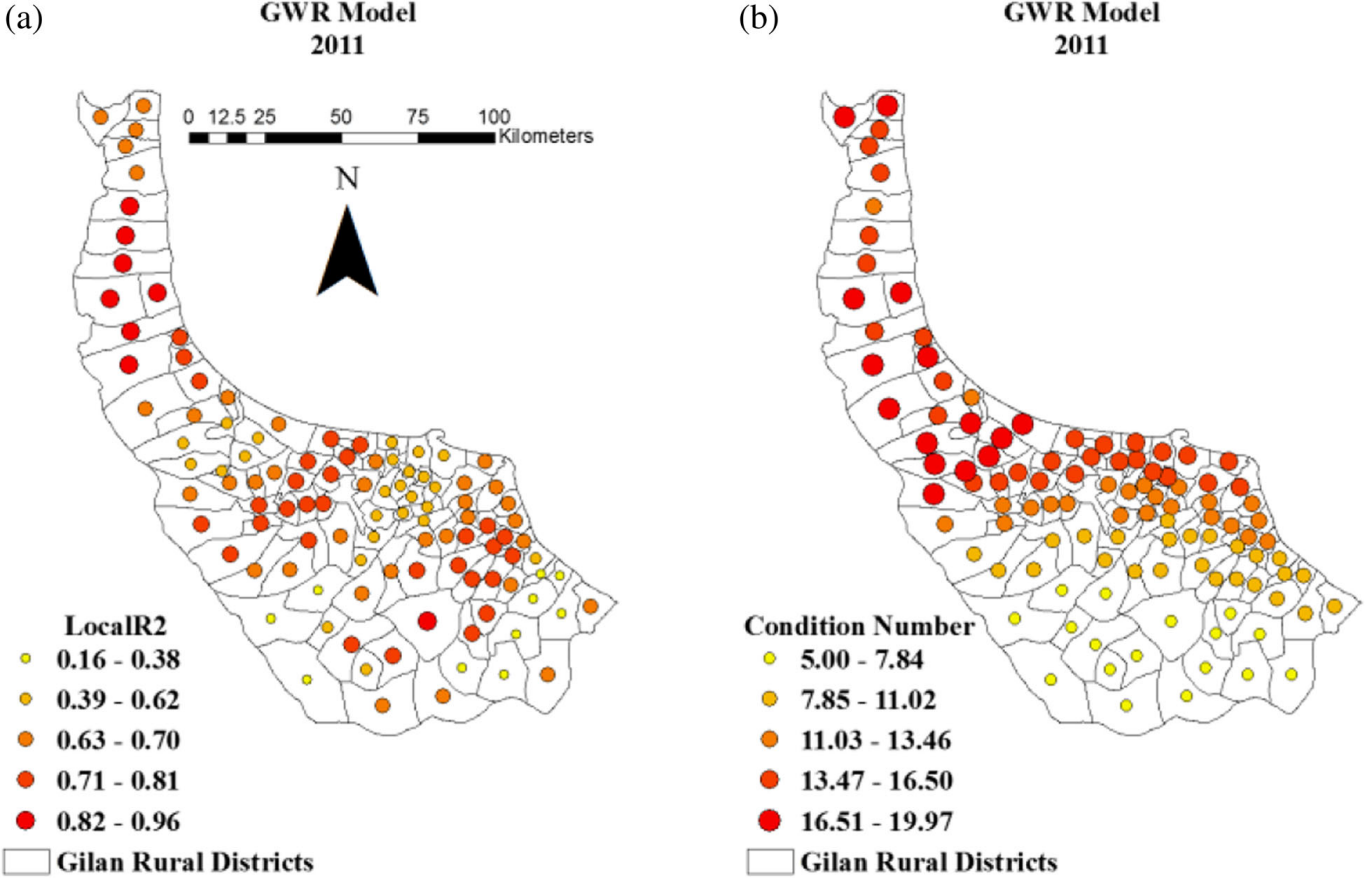
(**a**) Local R2 of GWR and (**b**) Condition number

Local variability of GWR model in each rural district is shown in Fig. 9.a. The size of dots in the map illustrates the prediction accuracy of GWR model in different rural districts. Local collinearity of GWR model is examined to evaluate the fitness of model via calculating condition numbers for each rural district (Fig. 9.b).

**Fig. 10:**
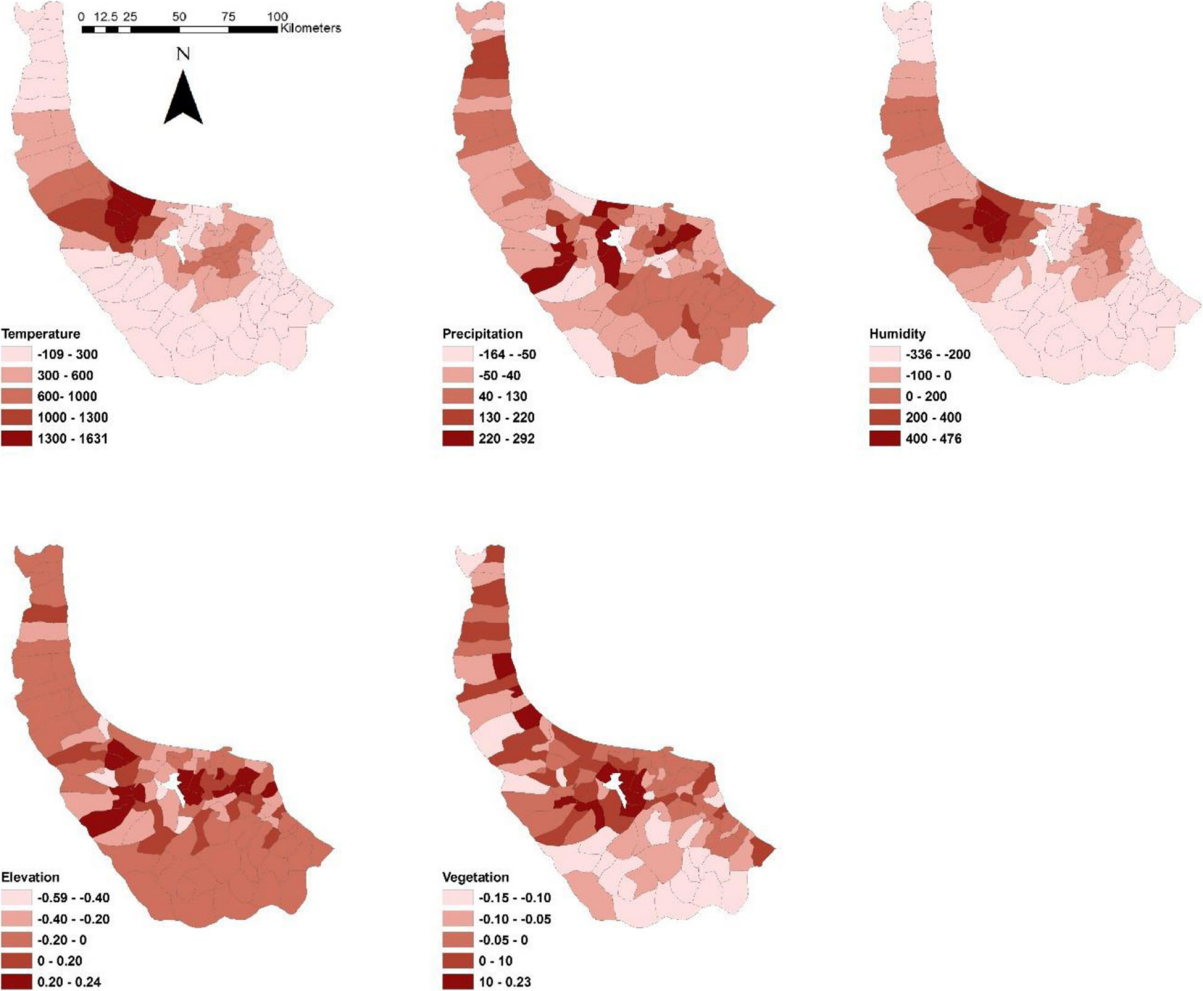
Coefficients of parameters obtained from GWR prediction model

The coefficients of GWR model are demonstrated in Fig. 10. Similarities are observed between coefficients of temperature and humidity with prediction map of 2011.

**Fig. 11:**
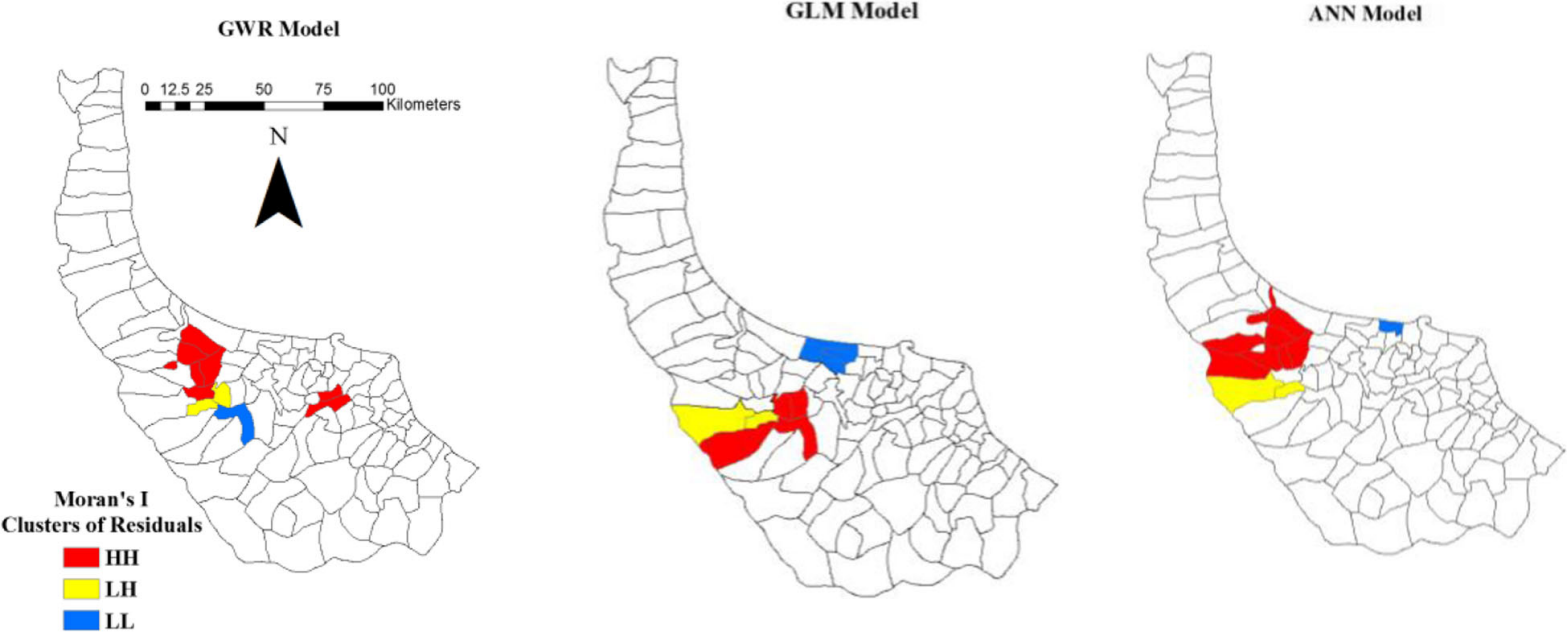
Spatial clusters of GWR, GLM and ANN residuals

Detected clusters at 95% significance level are demonstrated in Fig. 11 for GWR, GLM and ANN models. GWR, GLM and ANN models do not perform well in leptospirosis prediction in several districts.

## Discussion

During 2009–2011, reports of leptospirosis in Gilan revealed that it occurs in definite months and disappears for the remainder of the year. This periodic prevalence explains the relationship between leptospirosis cases and paddy season when workers start to work in paddy fields. This phenomenon is due to the fact that in paddy season when workers begin to plant or harvest rice, their contact with contaminated water or soil increases, and the possibility of disease prevalence increases. In Gilan, rice farming and livestock are popular amongst farmers because suitable climate contributes to the fertility of soil which is inevitable for farming, and the existence of many rural regions covered by grasslands and forests facilitates feeding animals. Considering that this job is physically demanding, the ratio of men to women workers is approximately 2 to 1 in 2009–2011, which confirms that men are more vulnerable to this disease and deserve more attention (Fig. 7.b). This fact prompted decision makers to carry out prevention programmes such as boosting the knowledge of workers by explaining the advantages of using gloves during work time or bandaging the wound as soon as it occurs. Knowledge and literacy are at low levels in rural districts, so such programmes led to a great decrease of disease reports (almost 1/3) in 2011 (Fig. 7.a).

Spatial modelling of leptospirosis would better clarify different aspects of this phenomenon. To model the disease, the correlation between input parameters should be investigated using the assumption of independence [70]. Correlation values vary from 0 (no correlation between two parameters) to 1 (maximum correlation between two parameters), and the closer the values are to 0, the more reliable they are as input in the model. Based on statistical studies about the assumption of independence, less than 0.70 correlation is acceptable [71]. Thus, two-tailed Pearson correlation as a common approach [72] is used in this study to calculate the correlation amongst all parameters. According to the obtained values, maximum correlation is between elevation and temperature parameters (0.33) with 0.005 significance level, and minimum is between vegetation and temperature (0.11) with 0.1 significance level. The results prove that all values are less than critical threshold (0.70) [71] and can be reliably utilized in spatial modelling of leptospirosis (Table 3).

In addition to assumption of independence, multicollinearity should be considered in spatial modelling [73]. Severe multicollinearity increases the variance estimation of coefficients and decreases the reliability of the model. VIF measures the intensity of multicollinearity amongst independent parameters [74]. Confirmed by statistical studies, VIF values of input parameters that are less than 10 are acceptable for entering the model [75]. Table 3 presents that the maximum calculated VIF values of parameters belong to vegetation parameter (2.71), and the minimum is acquired for precipitation parameter (1.17). All VIF values are less than 10, which proves acceptable multicollinearity amongst input parameters. According to the assumption of independence and VIF values, input parameters can be fed to GWR, GLM, SVM and ANN models for predicting leptospirosis distribution in this study.

The values of coefficients calculated for each parameter using GWR and GLM are presented in Table 4 and Table 5. GWR considers a different model for each rural district, so the coefficients of parameters vary across the study area. Slight changes in the range of elevation (D_2009_ = 0.17, D_2010_ = 0.73 and D_2011_ = 0.13) and vegetation (D_2009_ = 0.09, D_2010_ = 0.14 and D_2011_ = 0.16) reveal almost uniform and constant effect of these parameters. High values of temperature, precipitation and humidity range (1740.69, 321.64 and 812.94, respectively) show inconstant effects on diverse rural districts. Despite GWR and GLM models, ANN and SVM operate as black box. The coefficients of parameters cannot be calculated, but sensitivity analysis can be utilized for this issue. The results of sensitivity analysis are presented in Table 6 and Table 7, which show the effect of parameters on spatial modelling of leptospirosis distribution. According to four evaluation criteria, omission of temperature and humidity parameters decreases the fitness of the models, which confirms their importance in modelling the disease. Temperature and humidity do not directly affect leptospirosis distribution but provide appropriate circumstances for durability of leptospira and indirectly affect the prevalence of leptospirosis. Paddy fields are almost always located in rural districts with higher values of these parameters, and they are more vulnerable to the disease occurrence, as shown in Fig. 10, where coefficients are mapped for better understanding of the effect of parameters on different rural districts. Maps of coefficients of humidity and temperature are closer to prediction maps and reports of leptospirosis data in 2011. This finding proves that these two parameters play more important roles in the modelling and predicting leptospirosis.

### Prediction maps of GWR, GLM, SVM and ANN

The models clarify the fact that the disease prevalence occurs more in the central rural districts. The existing remarkable number of paddy fields and livestock activities, which leads people to more contact with the contaminated environment, can be the major reasons of this pattern. Given that leptospirosis is an occupational water-borne disease [76] and no paddy fields are in the southeast area of the province, the probability of the disease prevalence is negligible there. Visual comparison of the prediction maps shows that GWR, SVM and GLM models predict high disease prevalence in the central rural districts while the prediction of ANN model is less consistence with the reported cases of disease across the study area. Although SVM and GLM indicate satisfying results, GWR prediction map in 2011 is more similar to the map of leptospirosis data in 2011. Model predictions are statistically discussed in the “prediction evaluation” section.

A major advantage of GWR model is the presentation of local variability and local collinearity [77] which are not available in modelling with ANN, SVM and GLM. Local variability for each rural district shows the power of the model in different locations across the study area. Figure 9a demonstrates that GWR model performs more accurately on some rural districts with high local R^2^. The maximum value is 0.96, and the minimum is 0.16, but the overall R^2^ is 0.85 for the entire study area (Fig. 9a). The other issue is local collinearity, which is unavoidable in modelling and it has adverse effects on the estimation of coefficients. According to many studies, local collinearity of more than 30 indicates decreased reliability of results [78]. GWR shows local collinearity by measuring the condition number for each location. Condition numbers over 30 result in serious concern. Condition number measures how much the output value of the model can change for a small variation in the input of the model. Figure 9b indicates that the obtained condition number for each rural district is less than 20, and the local collinearity is negligible for the prediction of leptospirosis.

### Prediction evaluation

GWR, GLM, SVM and ANN models are trained by utilising the data of 2009 and 2010 to predict leptospirosis distribution in 2011. The results are compared with observations of leptospirosis (reported cases) in 2011. Four evaluation criteria, including R^2^, MAE, MSE and MRE, are employed to assess the results (Table 8). The values of R^2^ are 0.85, 0.78, 0.80 and 0.75 for GWR, GLM, SVM and ANN models, respectively. The values of MSE, MAE and MRE are calculated for GWR (0.050, 0.012 and 0.011), GLM (0.118, 0.052 and 0.017), SVM (0.103, 0.037 and 0.015) and ANN (0.137, 0.063 and 0.018). Needless to say, the lower the values of these criteria, the better the efficiency of the model. Hence, the performance of models in prediction of leptospirosis is GWR > SVM > GLM > ANN. This might be attributed to several reasons: The advantage of GWR as a weighted regression in modelling local variability and spatial heterogeneity, the nature of leptospirosis distribution varying across the study area locally, the superiority of SVM, as a supervised learning approach, in dealing with small classified datasets, the structure of GLM considering a polynomial with constant coefficients throughout the region and the shortcoming of ANN in handling small datasets.

**Table 8:** Evaluation results of GWR, GLM, SVM and ANN in modelling Leptospirosis

Approach	R^2^	MSE	MAE	MRE
GWR	0.85	0.050	0.012	0.011
GLM	0.78	0.118	0.052	0.017
ANN	0.75	0.137	0.063	0.018
SVM	0.80	0.103	0.037	0.015

### Spatial autocorrelation (Moran’s I) of residuals and significance level

Spatial autocorrelation in the residuals of model verifies weakness in some parts of the model [79]. In this study, weak but meaningful spatial autocorrelation is found in residuals. Environmental parameters model and predict the disease carefully, but the power of model is less in some regions. The capability of Moran’s I is verified in the investigation of residuals [80], so it is used in this study.

The results of Moran’s I are presented in Table 9. A greater convergence of Moran’s I to expected index indicates better performance of clustering [81]. In addition, z-score and *p*-value are criteria to determine the fitness of models. The lower value of *p*-value and the higher value of z-score elucidate that residuals of models are clustered in some rural districts. The values of Moran’s I, z-score and p-value are (0.2947, 0.3673 and 0.5406), (6.71, 7.63 and 12.01) and (0.0010, 0.0010 and 0.0012) for GWR, GLM and ANN respectively. Moran’s I of GWR is closer to Expected Index (− 0.0093) and the value of z-score is lower than GLM and ANN. It means GWR presents less deficiency in modelling and predicting leptospirosis. The result of spatial autocorrelation on residual for SVM is not presented in this part because SVM works with the label of classes.

**Table 9:** Results of Moran’s I for GWR, GLM and ANN residuals in 2009–2011

Method	Moran’s Index	Expected Index	z-score	*p*-value
GWR	0.2941	−0.0093	6.71	0.0010
GLM	0.3673	−0.0093	7.63	0.0010
ANN	0.5406	−0.0093	12.01	0.0012

Spatial clusters of GWR, GLM and ANN residuals obtained from Moran’s I approach are presented in Fig. 11. It illustrates the performance of models for prediction in various areas. High–High (HH) shows rural districts surrounded by neighbours with high spatial autocorrelation. Low–High (LH) indicates rural districts that have low spatial autocorrelation of residuals, but their neighbours have high values. Low–Low (LL) presents rural districts surrounded by neighbours with low spatial autocorrelation. Given the high spatial autocorrelation in residuals, HH clusters illustrate the rural districts where the models have lower performance in prediction of leptospirosis.

## Conclusion

Leptospirosis is predicted in this study utilizing GWR, SVM, GLM and ANN models. Five input parameters, including temperature, precipitation, humidity, elevation and vegetation are used in this study. Model predictions are investigated statistically and visually to understand the efficiency of used approaches. According to the results, the performance of the models is as follow: GWR > SVM > GLM > ANN. Also, spatial autocorrelation of residuals is used to investigate the deficiency of models. The results prove that GWR presents less deficiency in modelling and predicting leptospirosis. Additionally, based on coefficients of GWR and GLM parameters and sensitivity analysis of SVM and ANN, temperature and humidity have greater effects on the leptospirosis distribution. Moreover, analysis of coefficients shows that higher temperature and humidity coincide with higher disease occurrence in central regions. In contrast, the southeast rural districts have the lowest outbreaks due to lack of related occupations conducive to leptospirosis propagation. In a nutshell, utilizing useful approaches for prediction of leptospirosis can provide health managers and governments with sufficient information to set proper measures for controlling the disease prevalence across the study area.

Many researches including our study are limited based on data and model. As an analytical shortcoming of many disease studies, Modified Areal Unit Problem (MAUP) presents that scale of study is crucial in spatial analysis [82]. In this study, the results of leptospirosis prediction are acceptable at the rural district level, but this disease should be examined in other scales for better understanding the fitness of models. Disease data used in this study are based on the address of patients, whereas the exact locations of the disease occurrence are paddy fields. The paddy fields must be considered as the base level for more accurate analysis, but such data are not available in Iran. More social and epidemiologic parameters should be considered for more accurate prediction.

As future work, the model will be developed by considering socioepidemiologic parameters. Time series models such as Autoregressive Integrated Moving Average (ARIMA) and their comparison with geographically temporal weighted regression is also considered as future work.

## Supplementary information

**Additional file 1.** The results of trial and error approach for ANN. The results of trial and error approach for finding the optimal numbers of hidden layers and nodes in layers in final MLP architecture were presented.

## Data Availability

The datasets used and/or analyzed during the current study are available from the corresponding author on reasonable request.

## Abbreviations

AIC: Akaike Information Criterion
ANN: Artificial Neural Network
ARIMA: Autoregressive Integrated Moving Average
BIC: Bayesian Information Criterion
CV: Cross Validation
ENVI: Environment for Visualizing Images
FFNN: Feed-Forward Neural Networks
GIS: Geographical Information System
GLM: Generalized Linear Model
GWR: Geographically Weighted Regression
HC: Health Centre
IDW: Inverse Distance Weighting
MAE: Mean Absolute Error
MAUP: Modified Areal Unit Problem
MLP: Multilayer Perceptron
MODIS: The Moderate Resolution Imaging Spectroradiometer
MRE: Mean Relative Error
MSE: Mean Square Error
NASA: National Aeronautics and Space Administration
NDVI: Normalised Difference Vegetation Index
NHCN: National Health Care Network
NMHT: National Ministry of Health and Treatment of Iran
RBF: Radial Basis Function
SRTM: Shuttle Radar Topography Mission
SVM: Support Vector Machine
VIF: Variance Inflation Factor

## Footnotes

1 Enzyme-Linked ImmunoSorbent Assay

2 Inverse Distance Weighting

3 National Aeronautics and Space Administration

4 Shuttle Radar Topography Mission

5 Environment for Visualizing Images

6 The Moderate Resolution Imaging Spectroradiometer

7 http://gwr.maynoothuniversity.ie/gwr4-software/
