# Supplementary material for: Prediction mapping of human leptospirosis using ANN, GWR, SVM and GLM approaches
Source: BMC Infect Dis. 2019 Nov 13;19:971. doi: 10.1186/s12879-019-4580-4 (PMC6854714; doi:10.1186/s12879-019-4580-4)
Supplement: Supplementary file 1 — Additional file 1. The results of trial and error approach for ANN. The results of trial and error approach for finding the optimal numbers of hidden layers and nodes in layers in final MLP architecture were presented. [file 12879_2019_4580_MOESM1_ESM.docx]

| **Additional file 1** | | | | | | | | |
| --- | --- | --- | --- | --- | --- | --- | --- | --- |
| **No. of Hidden Layers** | **No. of Nodes in Layers** | | | | **R^2^** | **MSE** | **MAE** | **MRE** |
|  | **1** | **2** | **3** | **4** |  |  |  |  |
| **1** | **1** | - | - | - | 0.73 | 0.161 | 0.084 | 0.26 |
|  | … | - | - | - | … | … | … | … |
|  | **5** | - | - | - | **0.75** | **0.137** | **0.063** | **0.018** |
| **2** | 1 | 1 | - | - | 0.70 | 0.363 | 0.132 | 0.98 |
|  |  | … | - | - | … | … | … | … |
|  |  | 5 | - | - | 0.68 | 0.402 | 0.260 | 0.103 |
|  | … | … | - | - | … | … | … | … |
|  | **5** | 1 | - | - | 0.69 | 0.394 | 0.253 | 0.102 |
|  |  | … | - | - | … | … | … | … |
|  |  | **4** | - | - | **0.71** | **0.345** | **0.118** | **0.84** |
|  |  | 5 | - | - | 0.70 | 0.362 | 0.130 | 0.97 |
| **3** | **1** | **1** | 1 | - | 0.69 | 0.401 | 0.257 | 0.103 |
|  |  |  | … | - | … | … | … | … |
|  |  |  | **5** | - | **0.70** | **0.360** | **0.131** | **0.96** |
|  |  | … | … |  | … | … | … | … |
|  |  | 5 | 1 | - | 0.69 | 0.399 | 0.255 | 0.104 |
|  |  |  | … | - | … | … | … | … |
|  |  |  | 5 | - | 0.69 | 0.400 | 0.257 | 0.102 |
|  | … | … | … | - | … | … | … | … |
|  | 5 | 5 | 1 | - | 0.68 | 0.414 | 0.264 | 0.104 |
|  |  |  | … | - | … | … | … | … |
|  |  |  | 5 | - | 0.68 | 0.418 | 0.269 | 0.106 |
| **4** | 1 | 1 | 1 | 1 | 0.67 | 0.470 | 0.301 | 0.139 |
|  |  |  |  | … | … | … | … | … |
|  |  |  |  | 5 | 0.66 | 0.501 | 0.326 | 0.168 |
|  | … | … | … | … | … | … | … | … |
|  | **4** | **5** | **3** | 1 | 0.67 | 0.462 | 0.299 | 0.134 |
|  |  |  |  | **2** | **0.68** | **0.429** | **0.271** | **0.107** |
|  |  |  |  | … | … | … | … | … |
|  |  |  |  | 5 | 0.65 | 0.523 | 0.341 | 0.179 |
|  | ... | ... | ... | ... | **...** | **...** | **...** | **...** |
|  | 5 | 5 | 5 | 1 | 0.66 | 0.498 | 0.319 | 0.163 |
|  |  |  |  | … | … | … | … | … |
|  |  |  |  | 5 | 0.65 | 0.539 | 0.354 | 0.182 |
